# Trichorhinophalangeal syndrome type 1 (TRPS1) in breast pathology: diagnostic utility and pitfalls

**DOI:** 10.1186/s13000-025-01623-4

**Published:** 2025-03-01

**Authors:** Atif Ali Hashmi, Edi Brogi, Hannah Y. Wen

**Affiliations:** Department of Pathology and Laboratory Medicine, Memorial Sloan Kettering Cancer Center, 1275 York Avenue, New York, NY 10065 USA

**Keywords:** TRPS1, GATA3, SOX10, Breast markers, Metastatic breast carcinoma, Triple negative breast cancer

## Abstract

**Introduction:**

Breast cancer, especially triple-negative breast cancer (TNBC), lacks sensitive and specific diagnostic markers that can reliably differentiate it from carcinomas of other origins. TRPS1 is a relatively new immunohistochemical (IHC) marker that has demonstrated higher sensitivity in breast cancer, including TNBC. However, with the increasing use of this marker, broader immunoreactivity has been observed. This study aims to evaluate the utility of TRPS1 for establishing carcinoma of mammary origin. We compared the diagnostic sensitivity and specificity of TRPS1 with that of other IHC markers (GATA3 and SOX10).

**Methods:**

In this retrospective study, we reviewed TRPS1 IHC performed at our center between 07/2022 and 06/2024, to evaluate the expression of TRPS1 in breast carcinoma (primary and distant metastasis) and in other malignancies. The sensitivity and specificity of TRPS1 in determining carcinoma of breast origin were compared with those of GATA3 and SOX10.

**Results:**

The study cohort comprised 106 cases, including 17 cases at the primary site, and 89 samples of distant metastasis. After correlation with morphology, immunophenotype and molecular studies, 94 cases (88.7%) were characterized as breast primary (37.9% ER+/HER2neu-, 4.6% ER-/HER2neu+, 1.1% ER+/HER2neu+, 56.3% TNBC), whereas 12 (11.3%) were non-breast primary. The non-breast primary sites included lung, bladder, Mullerian, and gastrointestinal. The sensitivity and specificity of TRPS1 were 93.6% and 58.3%, respectively. Conversely, GATA3 demonstrated a sensitivity and specificity of 76.9% and 66.7%, respectively. SOX10 exhibited the lowest sensitivity at 47.9%, but with the highest specificity at 100%. There were three cases of metastatic breast carcinoma (sites: bladder, lung, and bone), where TRPS1 was the only positive marker, whereas GATA3 and SOX10 were negative. TRPS1 showed a higher positivity rate (92.0%) in TNBC compared to GATA3 (63.4%) and SOX10 (56.7%). TRPS1 expression was also observed in other tumor types, including carcinoma of Mullerian origin, bladder, and lung, limiting its utility in the differential diagnosis.

**Conclusion:**

Our study demonstrated a higher sensitivity of TRPS1 expression in establishing carcinoma of breast origin compared with GATA3 and SOX10, consistent with previous reported studies. However, the specificity of TRPS1 was lower than that of GATA3 and SOX10. These findings suggest that while TRPS1 can be used as a reliable marker for breast cancer, its expression in other tumor types should be carefully interpreted to avoid diagnostic pitfalls.

## Introduction

Breast cancer is a heterogenous disease. For hormone receptor negative breast cancer, there is no sensitive and specific marker to definitively establish a diagnosis of carcinoma of mammary origin, especially in patients presenting with metastatic disease. The commonly used IHC markers to support a diagnosis of a carcinoma of breast primary include mammaglobin, gross cystic disease fluid protein 15 (GCDFP-15), GATA binding protein 3 (GATA3), and SRY-related HMG box 10 (SOX10), in addition to hormone receptors [1]. The sensitivity of these markers differs depending on histologic and intrinsic breast cancer subtypes. Poorly differentiated breast carcinoma is more likely to lose the expression of several markers, such as GCDFP-15 and mammaglobin [2]. GATA3 and SOX10 are useful markers; however, they are not specific for breast cancer. GATA3 is also expressed in urothelial carcinoma, skin adnexal tumors, paraganglioma, T-cell hematopoietic malignancies, among others [3]. Similarly, SOX10 is positive in melanoma and some soft tissue tumors [4].

Trichorhinophalangeal syndrome type 1 (TRPS1) is a GATA family of zinc transcription factors implicated in breast cancer carcinogenesis and involved in cancer cell survival. Studies have shown the high sensitivity of TRPS1 in estrogen receptor (ER)-positive (98%), human epidermal growth factor receptor 2 (HER2neu)-positive (87%), and triple negative breast cancer (TNBC) (86%), which is higher than other breast markers, including GATA3 [5]. Like GATA3, TRPS1 is expressed in cutaneous and skin adnexal tumors [6]. Unlike GATA3, TRPS1 is not expressed in urothelial carcinoma [5]. In our practice, as we increasingly incorporate TRPS1 in our immunohistochemical (IHC) workup, we have observed TRPS1 expression in a variety of malignancies beyond those of mammary origin. In this study, we conducted a retrospective review of TRPS1 IHC performed at our center since it was introduced in 2022. The aims were (1) to evaluate the sensitivity and specificity of TRPS1 in establishing carcinoma of breast origin; (2) to demonstrate its expression in carcinomas of other sites and highlighting the importance of interpretation with caution.

## Methods

### Case selection

This retrospective study was approved by the institutional review board. The institution pathology database was searched to identify surgical pathology cases in which TRPS1 IHC was performed at our center in the diagnostic work-up between July 2022 and June 2024. Clinicopathologic characteristics were extracted from the pathology report and medical record. Molecular testing by MSK-IMPACT was conducted in some cases when immunohistochemistry results were inconclusive to determine the origin of the metastatic carcinoma.

### Immunohistochemistry

**Fig. 1:**
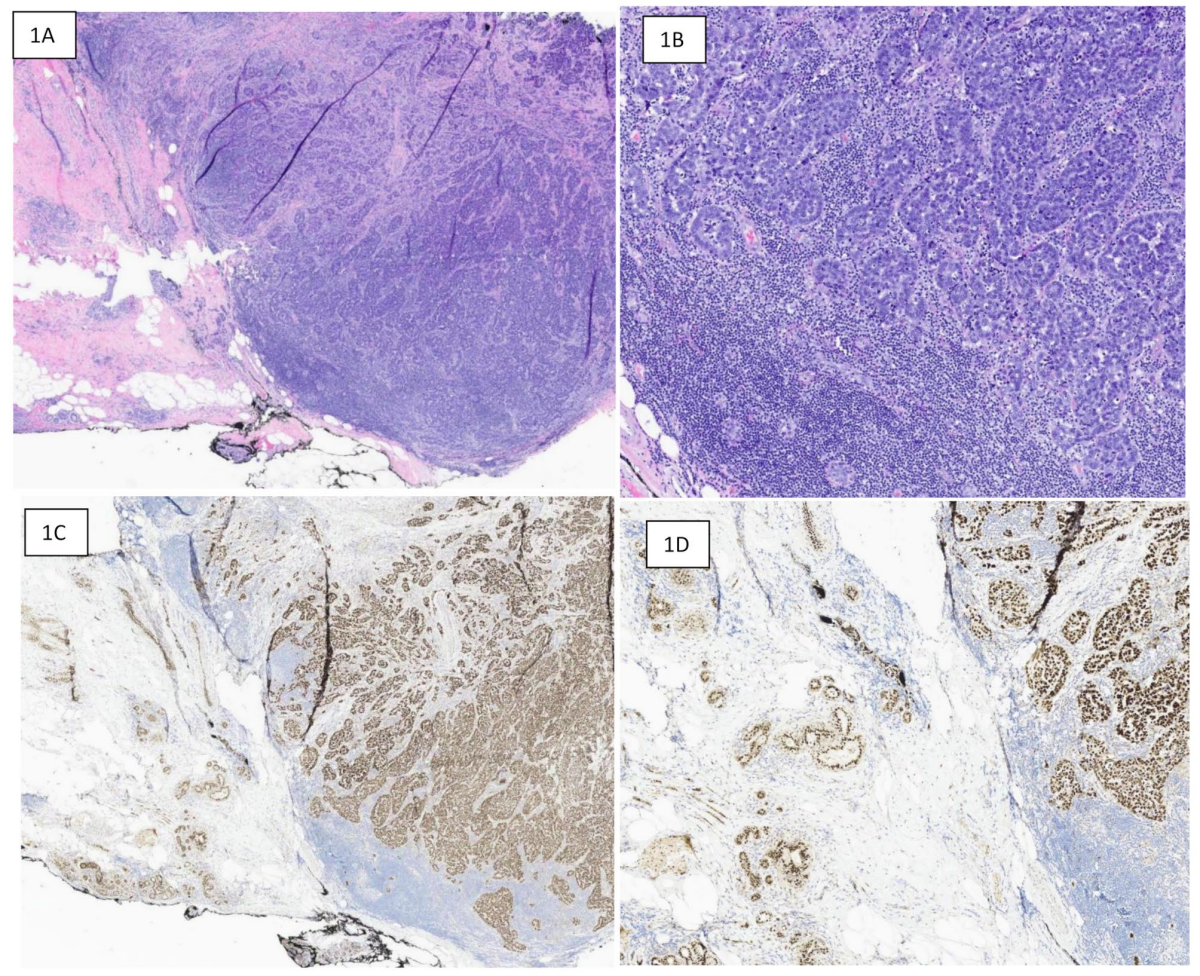
Poorly differentiated primary triple-negative breast carcinoma with TRPS1 expression. **1A**: Low power hematoxylin and eosin (H & E)-stained section showing poorly differentiated carcinoma with dense background inflammatory infiltrate and a relatively circumscribed border. **1B**: Higher power view (H & E)-section showing high grade solid pattern tumor growth. **1C**: TRPS1 (low power) showing diffuse strong positive staining in both tumor cells and background benign normal breast ducts and lobules. **1D**: TRPS1 (high power) showing nuclear positivity in both tumor (right) and normal breast parenchyma (left), serving as positive internal control

IHC staining for TRPS1 was performed using rabbit monoclonal antibody (clone EPR171671, Abcam) according to manufacturer’s protocol. Nuclear staining of tumor cells was considered positive. As there is no standard cut off for positive TRPS1 expression, weak to moderate staining in over 10% tumor cells was taken as positive. Conversely, strong nuclear staining in any tumor of tumor cells was considered positive. Majority of cases of our cohort showed diffuse positive expression. Positive and negative controls were run in parallel. In addition, nuclear staining of normal breast ducts and lobules serve as positive internal control (Fig. 1).

Immunohistochemical staining for GATA3 (clone L50-823; Biocare Medical) and SOX10 (clone BC34; Biocare Medical) were also performed in most cases. GCDFP15 and mammaglobin immunostaining was performed in a few cases. Biomarkers including ER, PR, HER2/neu, and androgen receptor (AR), were also assessed in most cases. Moreover, thyroid transcription factor 1 (TTF1), p40, PAX8, and other site-specific immunostaining was performed as necessary to confirm the primary origin. Our study also included bone samples with metastatic carcinoma. As decalcification may hinder the IHC processing, as a general practice at our institution, when bone samples with a clinical suspicion of metastatic carcinoma are submitted, any soft tissue is separated from the harder tissue material and processed separately without decalcification. The rest of the material is decalcified using EDTA bone decalcification protocol.

### Statistical analysis

The data were analyzed using the Statistical Package for Social Science (SPSS, Version 26.0; IBM Inc.). Sensitivity, specificity, positive predictive value (PPV), negative predictive value (NPV), and diagnostic accuracy were calculated for TRPS1, GATA3 and SOX10 by 2 × 2 tables with primary breast origin confirmed by morphologic correlation, immunophenotype, and in some cases, molecular analysis.

## Results

We identified 106 cases in which TRPS1 IHC was performed at the time of the diagnosis. Of these, TRPS1 was employed in the evaluation of 17 breast specimens to distinguish whether the tumor was a primary breast carcinoma (*n* = 14) or an extra-mammary metastasis to the breast (*n* = 3). In the remaining 89 cases, TRPS1 IHC was utilized in the diagnostic work-up for distant metastasis to determine whether the metastatic carcinoma was breast origin (*n* = 80) or from other sites (*n* = 9).

Table 1 shows the descriptive statistics of the study cohort in the primary setting (*n* = 17). There were 14 breast and 3 primary lung specimens in which TRPS1 was employed as a diagnostic workup. The mean age at diagnosis was 60.35 ± 10.65. Most breast tumors were ductal (*n* = 12, 85.7%), and high grade (*n* = 12, 85.7%).

**Table 1:** Descriptive statistics of study cohort in primary setting (*n* = 17)

Clinicopathological features	Primary
**Age (years)**	
Mean ± SD	60.35 ± 10.65
**Age groups**	
≤ 50 years	3 (17.6)
> 50 years	14 (82.4)
**Gender**	
Male	0 (0)
Female	17 (100)
Site of biopsy*	
Breast	14 (82.4)
Lung	3 (17.6)
**Primary breast tumor type (n = 14)**	
Ductal	12 (85.7)
Others	2 (14.2)
**Primary breast tumor grade (n = 14)**	
Low-intermediate	2 (14.3)
High	12 (85.7)

*There were 14 cases where TRPS1 was applied for confirming primary breast in breast biopsies. Similarly, in 3 cases of lung biopsy, the tumor was established as lung carcinoma, therefore included in primary cohort

Among metastatic cohort (*n* = 89), lung (*n* = 39, 43.8%), followed by bone (*n* = 13, 14.6%) were the most common biopsy sites. In 80 (89.9%) cases the primary origin of the tumor was breast, with lung being the second most common origin site (*n* = 4, 4.5%). Most breast tumors were high grade (64.3%), as presented in Table 2.

**Table 2:** Descriptive statistics of study cohort in metastatic setting (*n* = 89)

Clinicopathological parameters	*n* (%)
**Age (years)**	
Mean ± SD	58.16 ± 14.45
**Age groups**	
≤ 50 years	29 (32.6)
> 50 years	60 (67.4)
**Gender**	
Male	2 (2.2)
Female	87 (97.8)
**Site of biopsy**	
Lung	39 (43.82)
Bone	13 (14.61)
Axillary lymph node	3 (3.37)
Bladder	2 (2.25)
Liver	5 (5.62)
Mediastinal lymph node	9 (10.11)
Pleura	3 (3.37)
Skin	2 (2.25)
Others	13 (14.60)
**Primary origin of tumor**	
Breast	80 (89.9)
Bladder	2 (2.2)
Lung	4 (4.5)
Mullerian	2 (2.2)
Gastrointestinal	1 (1.1)
**History of breast cancer**	
Yes	77 (86.5)
No	12 (13.5)
Primary breast tumor type (n = 70) *	
Ductal	58 (82.9)
Lobular	2 (2.9)
Metaplastic	6 (8.6)
Micropapillary	2 (2.9)
Others	3 (4.2)
Primary breast tumor grade (n = 70) *	
Low-intermediate	25 (35.7)
High	45 (64.3)

*Tumor type/grade for review were only available in 70 out of 80 cases where the primary origin was breast in metastatic setting

Table 3 shows IHC markers expression in cases with breast as primary site of origin (*n* = 94), as established after morphological correlation/IHC/molecular studies. TRPS1 showed highest positivity (93.6%), compared with other breast-specific markers (GATA3: 76.9%; SOX10: 47.9%). ER, PR, HER2neu and AR positivity were 41.1%, 20.5%, 5.5%, and 45.5%, respectively.

**Table 3:** Immuno-marker expression in cases with breast as primary site of origin (*n* = 94)

Expression	Immunomarker, *n* (%)									
	TRPS1 (*n* = 94)	GATA3 (*n* = 78)	SOX10 (*n* = 48)	ER (*n* = 90)	PR (*n* = 88)	AR (*n* = 33)	Her2neu (*n* = 86)	TTF1 (*n* = 36)	P40 (*n* = 24)	PAX8 (*n* = 7)
Positive	88 (93.6)	60 (76.9)	23 (47.9)	37 (41.1)	18 (20.5)	15 (45.5)	5 (5.8)	1 (2.8)	5 (20.8)	0 (0)
Negative	6 (6.4)	18 (23.1)	25 (52.1)	53 (58.9)	70 (79.5)	18 (54.5)	81 (94.2)	35 (97.2)	19 (79.2)	7 (100)

There were 12 cases in which the primary origin of the tumor was non-breast. Notably, 5 (41.7%) cases showed TRPS1 expression, whereas this frequency was low with other IHC markers (GATA3: 33.3%; SOX10: 0%), as shown in Table 4.

**Table 4:** Immuno-marker expression in cases with non-breast primary (*n* = 12)

Expression	Immunomarker, *n* (%)									
	TRPS1 (*n* = 12)	GATA3 (*n* = 9)	SOX10 (*n* = 8)	ER (*n* = 7)	PR (*n* = 6)	AR (*n* = 3)	Her2neu (*n* = 3)	TTF1 (*n* = 7)	P40 (*n* = 3)	PAX8 (*n* = 4)
Positive	5 (41.7)	3 (33.3)	0 (0)	1 (14.3)	1 (16.7)	0 (0)	0 (0)	5 (71.4)	1 (33.3)	1 (25)
Negative	7 (58.3)	6 (66.7)	8 (100)	6 (85.7)	5 (83.3)	3 (100)	3 (100)	2 (28.6)	2 (66.7)	3 (75)

Table 5 shows the diagnostic accuracy of TRPS1, GATA3 and SOX10 in establishing breast as primary site of origin in both primary and metastatic settings. The sensitivity and specificity of TRPS1 were 93.6% and 58.3%, respectively. Conversely, GATA3 demonstrated a sensitivity and specificity of 76.9% and 66.7%, respectively. SOX10 exhibited the lowest sensitivity at 47.9%, but with the highest specificity at 100%.

**Table 5:** Diagnostic accuracy of TRPS1, GATA3 and SOX10 for the diagnosis of breast carcinoma

Immunomarker	Expression	Primary origin of cancer		Sensitivity	Specificity	PPV	NPV	Diagnostic accuracy
		Breast	Others					
TRPS1	Positive	88	5	93.6%	58.3%	94.6%	53.8%	89.62%
	Negative	6	7					
GATA3	Positive	60	3	76.9%	66.7%	95.2%	25%	75.86%
	Negative	18	6					
SOX10	Positive	23	0	47.9%	100%	100%	24.2%	55.35%
	Negative	25	8					

PPV: positive predictive value; NPV: negative predictive value

**Fig. 2:**
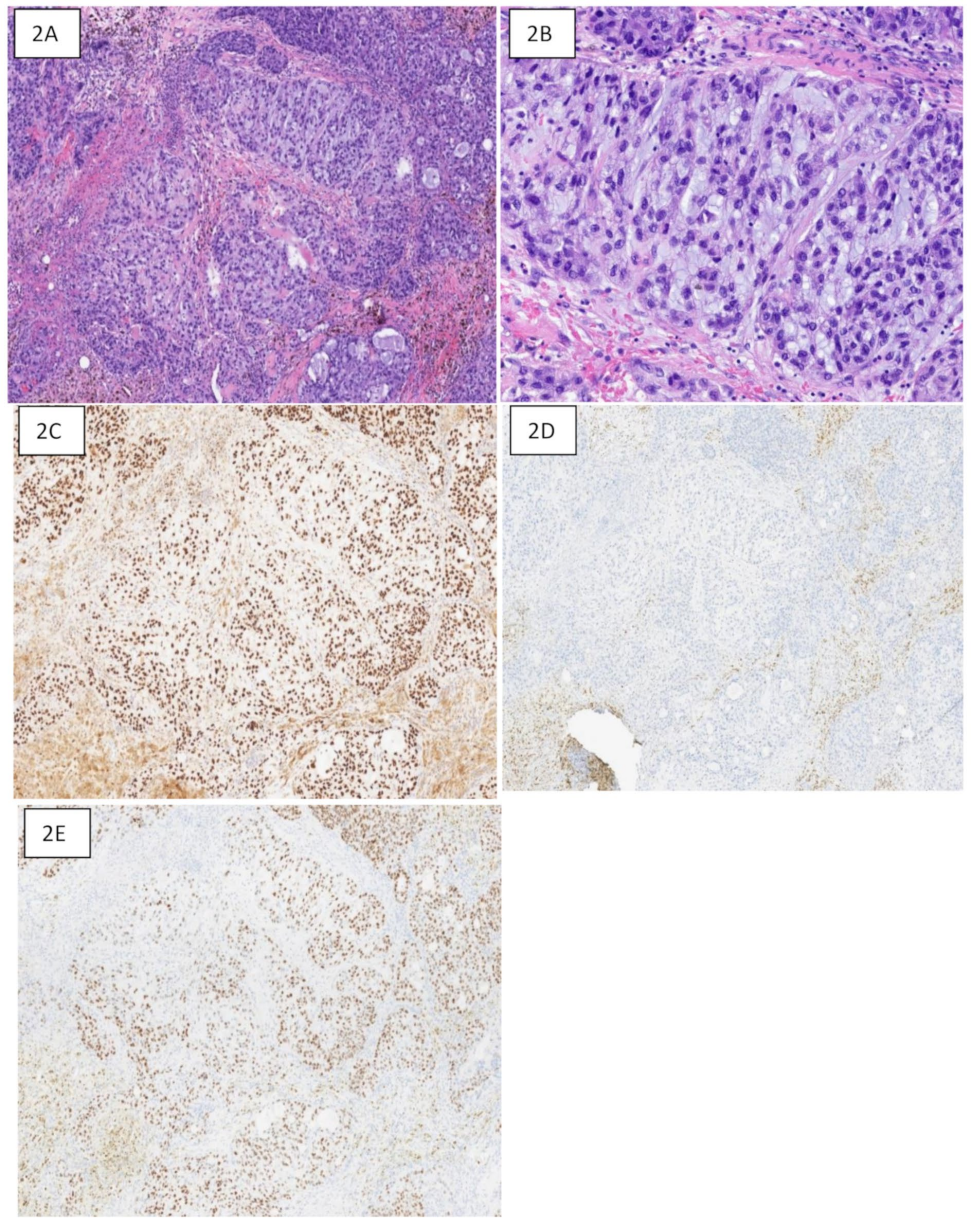
Metaplastic primary breast carcinoma with TPRS1, GATA3 and SOX10 immunostains. **2A**: Hematoxylin and eosin (H & E)-stained section at 50X magnification showing poorly differentiated carcinoma with matrix-producing stroma consistent with metaplastic breast carcinoma. **2B**: H & E-stained section at 100x showing tumor cells with matrix-producing stroma in the background. **2C**: TRPS1 immunostaining at 50X magnification depicting strong diffuse nuclear positivity in tumor cells. **2D**: GATA3 immunostaining revealing negative nuclear expression. Background cytoplasmic staining is seen in stroma. **2E**: SOX10 immunostaining at same magnification showing patching moderate nuclear staining in tumor cells

There were three cases of metastatic breast carcinoma (sites: bladder, lung, and bone), where TRPS1 was the only positive marker, whereas GATA3 and SOX10 were negative. Figure 2 shows a case of metaplastic breast carcinoma with strong TRPS1 staining. GATA3 staining was negative and SOX10 staining was patchy.

**Fig. 3:**
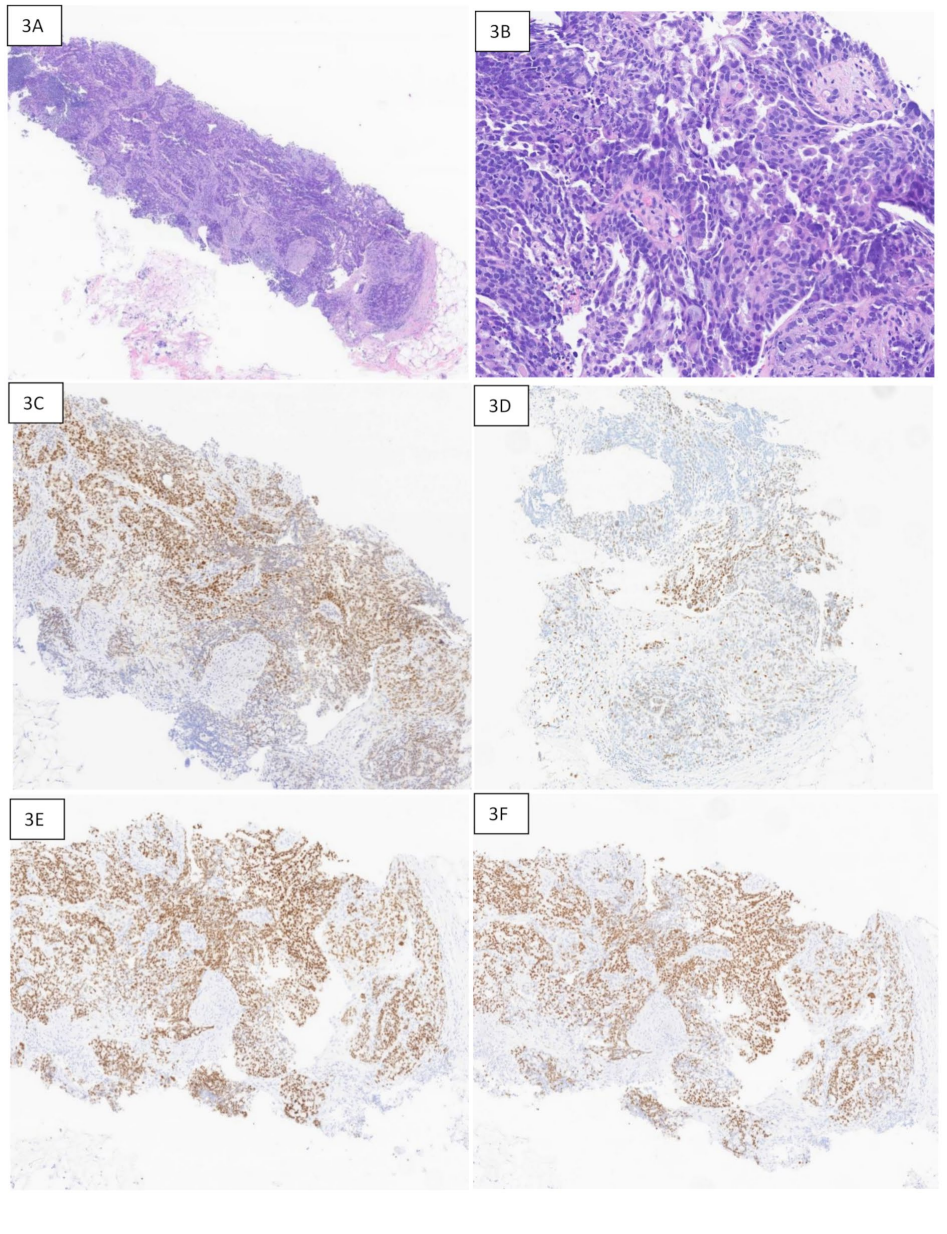
Metastatic serous carcinoma of tubo-ovarian origin to breast with TPRS1, GATA3, PAX8, and WT1 immunostaining. **3A**: H & E-stained section at 20X magnification showing a breast core with invasive carcinoma. **3B**: H & E-stained section at 50X revealing papillary growth pattern. **3C**: TRPS1 immunostaining at 50X magnification showing moderate nuclear staining. **3D**: GATA3 immunostaining revealing patchy moderate staining. **3E**: PAX8 immunostaining at 50X depicting strong nuclear staining. **3F**: WT1 immunostaining at 50X revealing strong diffuse nuclear staining consistent with tubo-ovarian primary origin

**Fig. 4:**
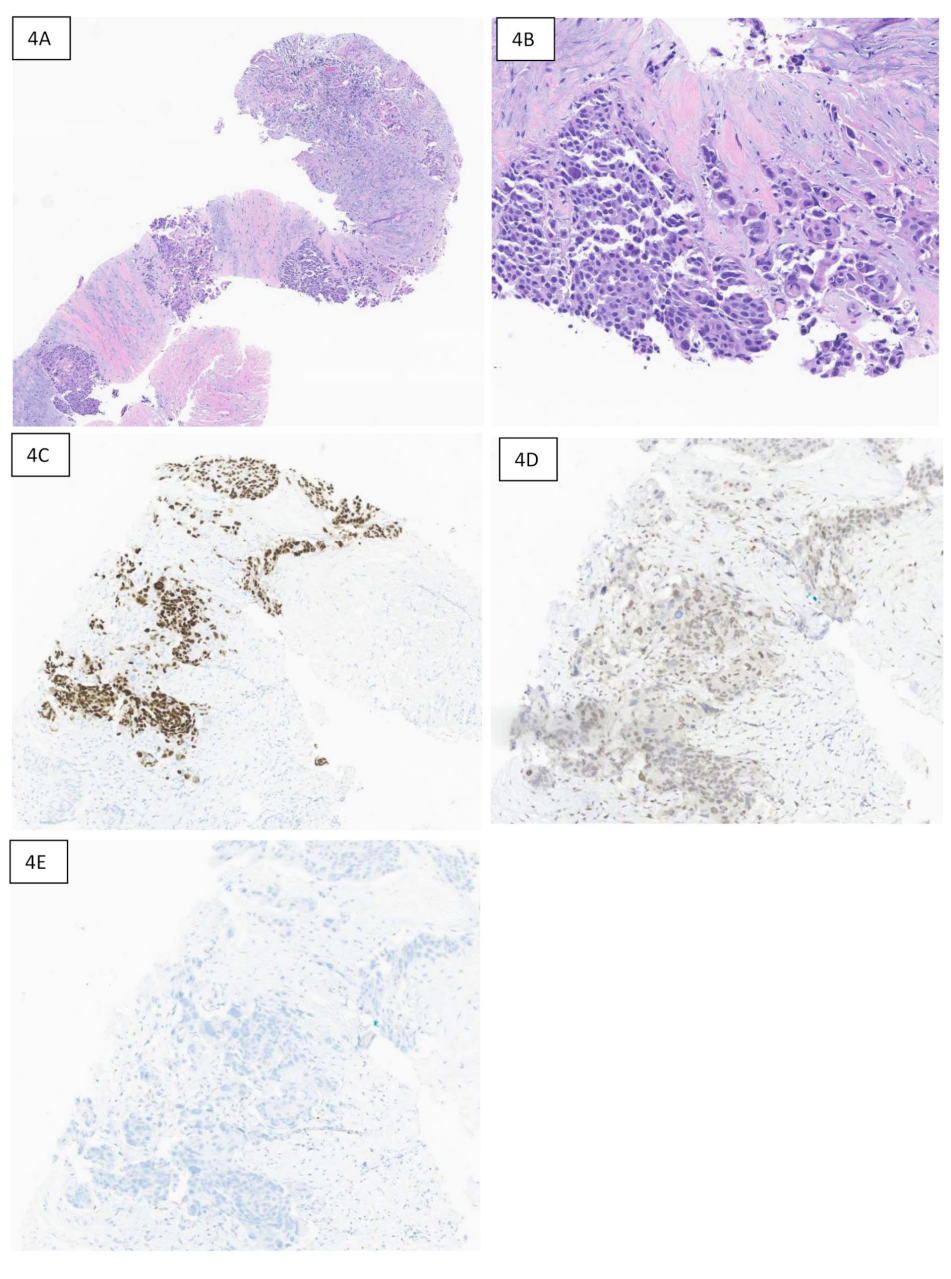
Metastatic urothelial carcinoma to the pelvis, with P63, TRPS1, and GATA3 immunostaining. **4A**: H & E-stained section at 50X showing fibro-collagenous tissue with nests and clusters of metastatic carcinoma. **4B**: H & E-stained section at 100X showing epithelioid tumor cells. **4C**: P63 immunostaining revealing strong diffuse nuclear positivity. **4D**: TRPS1 showing patchy weak nuclear positivity. **4E**: GATA3 immunostaining showing negative staining in tumor cells

Conversely, as noted in Table 5, the specificity of TRPS1 for breast carcinoma was low. TRPS1 positivity was noted in five cases of non-breast primary tumors (2 lung, 1 bladder, 1 tubo-ovarian, and 1 endometrial). The two Mullerian origin tumors (endometrial and tubo-ovarian) were also positive with GATA3 immunostain. In one of them (endometrial) SOX10 was performed and was negative. Figure 3 presents a case of high-grade serous carcinoma of tubo-ovarian origin metastatic to breast. Moderate nuclear staining for TRPS1 was noted in cancer cells. Similarly, Fig. 4 shows a case of metastatic urothelial carcinoma to pelvis, with patchy (weak) TRPS1 positivity.

Table 6 shows the association between TRPS1 expression and pathological parameters and biomarker status. No significant association of TRPS1 expression was noted with biomarker profile and tumor features (*p* > 0.05).

**Table 6:** Association of TRPS1 expression with biomarker profile and pathological features

Biomarkers and pathological features	TRPS1, *n* (%)		*p*-value
	Positive	Negative	
**ER (n = 97)**			
Positive	37 (42.0)	1 (11.1)	0.085
Negative	51 (58.0)	8 (88.9)	
**PR (n = 94)**			
Positive	19 (21.8)	0 (0)	0.338
Negative	68 (78.2)	7 (100)	
**HER2neu (n = 89)**			
Positive	5 (6)	0 (0)	1.000
Negative	79 (94)	5 (100)	
Tumor type (n = 84) *			
Ductal	65 (83.3)	5 (83.3)	0.194
Lobular	2 (2.6)	0 (0)	
Metaplastic	7 (9)	0 (0)	
Micropapillary	3 (3.8)	0 (0)	
Others	1 (1.3)	1 (16.7)	
Tumor grade (n = 84) *			
Low-intermediate	24 (30.8)	3 (50)	0.381
High	54 (69.2)	3 (50)	

Fisher’s exact test was applied. *Tumor type/grade for review were only available in 84 out of 94 cases where the primary origin was breast (overall)

Table 7 shows the comparison of TRPS1 positivity rate in tumors of breast origin (either primary or metastatic with other breast specific markers with respect to tumor type. It is notable that TRPS1 positivity was 100% in all special type breast cancers in this cohort, including metaplastic carcinoma, whereas the positivity rate of metaplastic carcinoma was low with SOX10 and GATA3.

**Table 7:** Comparison of TRPS1, SOX10, and GATA3 positivity with respect to tumor type (*n* = 84)

Tumor type	*n*/total number performed (%)		
	TRPS1	SOX10	GATA3
Ductal, *n* = 70	65/70 (92.9)	17/37 (45.9)	48/58 (82.8)
Lobular, *n* = 2	2/2 (100)	-	0/1 (0)
Metaplastic, *n* = 7	7/7 (100)	4/6 (66.7)	3/7 (42.9)
Micropapillary, *n* = 3	3/3 (100)	1/1 (100)	2/2 (100)
Others, *n* = 2	1/2 (100)	1/1 (100)	0/1 (0)

Table 8 compares the positivity of TRPS1, SOX10, and GATA3 in breast-origin tumors according to tumor grade. TRPS1 shows higher positivity than GATA3 and SOX10, especially in high-grade tumors. It is also worth noting that the sensitivity of SOX10 is low in well-differentiated/low-grade breast carcinoma.

**Table 8:** Comparison of TRPS1, SOX10, and GATA3 positivity with respect to tumor grade (*n* = 84)

Tumor grade	*n*/total number performed (%)		
	TRPS1	SOX10	GATA3
Low-intermediate, *n* = 27	24/27 (88.9)	2/11 (18.2)	17/22(77.3)
High, *n* = 57	54/57 (94.7)	21/34 (61.8)	36/47 (76.6)

All biomarkers (ER, PR, and HER2neu) were performed in 87 cases, out of which 37.9% (33/87) were ER+/HER2neu-, 1.1% (1/87) were ER+/HER2neu+, 4.6% (4/87) were ER-/HER2neu+, while 56.3% (40/87) were triple-negative. Table 9 depicts the comparison of immunomarker positivity with respect to receptor (ER/PR/Her2neu) status. TRPS1 showed a higher positivity rate, especially in TNBCs. The sensitivity of GATA3 is comparable to that of TRPS1 in hormone receptor-positive tumors, whereas its positivity is low in TNBC. Conversely, SOX10 positivity is low in ER/PR-positive tumors.

**Table 9:** Comparison of TRPS1, SOX10, and GATA3 positivity with respect to biomarker status (*n* = 87)

Receptor status	*n*/total number performed (%)		
	TRPS1	SOX10	GATA3
ER/PR+, HER2neu-; *n* = 33	32/33 (97)	3/11 (27.3)	26/27 (96.3)
ER/PR+, HER2neu+; *n* = 1	1/1 (100)	-	1/1 (100)
ER/PR-, HER2neu+; *n* = 4	4/4 (100)	0/3 (0)	2/4 (50)
Triple-negative; *n* = 49	45/49 (91.8)	18/30 (60)	26/40 (65)

## Discussion

In this study, we found a high sensitivity of TRPS1 for determining primary breast origin in both primary and metastatic breast cancer; however, its specificity was lower than that of other commonly used markers, including GATA3 and SOX10, limiting its use particularly in the metastatic setting. We also found that TRPS1 positivity was higher than GATA3 and SOX10 in metaplastic and TNBCs. Conversely, TRPS1 positivity was noted in metastatic cancers of lung, bladder and Mullerian origin, making its usage as a sole breast marker questionable.

Breast cancer has a variable clinical behavior with a high propensity to metastasize. Breast cancer can metastasize to a wide variety of body sites, even after decades of primary breast cancer diagnosis [7, 8]. Morphologically, breast cancer mimics cancers of different origins, especially lung cancer, and it is sometimes very challenging to differentiate between the two types, particularly when the patient has a history of more than one cancer. In addition, although breast is not a common site of metastatic cancer, sometimes when another cancer metastasizes to the breast, there is a high propensity for misdiagnosis. In this study, we determined the primary origin of cancer via both morphologic correlations, IHC workup and molecular analysis (in some cases) and then compared the sensitivity and specificity of TRPS1 with other commonly used breast-specific markers.

### High TRPS1 sensitivity in triple-negative and metaplastic breast carcinoma

In metastatic setting, hormone receptor (ER/PR) positivity helps in establishing primary breast origin, along with other immunomarkers. A real diagnostic dilemma arises in the setting of TNBC with distant metastasis, with the role of immunohistochemistry, especially when molecular facilities are not available. We found higher positivity for TRPS1 in TNBC than for GATA3 and SOX10 (93.6% for TRPS1 vs. 76.9% and 47.9% for GATA3 and SOX10, respectively). These findings are comparable with those of the existing literature. Yoon et al. compared the positivity of these three immunomarkers and found that the positivity of TRPS1 was 99% for triple-negative ductal carcinoma vs. 63% and 74% for GATA3 and SOX10, respectively [9]. Similarly, Ai et al. studied TRPS1 expression at both molecular/mRNA and protein expression/IHC level. They found that TRPS1 was highly expressed at mRNA level across all breast cancer subtypes among 31 different solid tumors studied. They also showed that TRPS1 was equally expressed in luminal A, luminal B, HER2neu and basal-like/TNBC, unlike GATA3, expression of which was preferentially low in basal-like/TNBC. On IHC level they found that although TRPS1 and GATA3 expression were comparable in hormone-positive and HER2neu-positive cancers, however the expression of TRPS1 was higher in TNBC, particularly metaplastic carcinoma compared with GATA3 (86% vs. 21%) [5]. Concordant with these findings, we noted a considerably higher positivity for TRPS1 in metaplastic breast carcinoma (100%) compared with GATA3 (42.9%) and SOX10 (66.7%). These findings are supported by other studies [10].

Similar findings were noted in other studies. Parkinson et al. found a higher positivity for TRPS1 than GATA3 in metaplastic breast carcinoma (91% for TRPS1 vs. 55.2% for GATA3) [11]. It is also worth considering that TRPS1 is a marker of cartilage and bone development, and therefore, its utility is compromised when the differential diagnosis is primary or metastatic sarcoma or malignant phyllodes tumor with osteochondroid differentiation [12]. Therefore, TRPS1 may not be used as a diagnostic tool to differentiate between metaplastic carcinoma and malignant phyllodes tumor. Moreover, TRPS1 was shown to be expressed in skin tumors (including squamous cell carcinoma) [13], therefore, it is not clear if TRPS1 positivity in metaplastic cancers represents a true breast specific expression or just because of squamous/mesenchymal differentiation.

### Low specificity of TRPS1 compared with other immunomarkers

Despite overall better diagnostic accuracy, the specificity of TRPS1 was lower than that of GATA3 and SOX10. Tumors of Müllerian origin were found to have TRPS1 positivity. We had two cases of Mullerian origin tumors that showed TRPS1 positivity. The first case was that of high-grade serous carcinoma of tubo-ovarian origin metastatic to the breast, that showed TRPS1 positivity. Strong diffuse PAX8 and WT1 positivity, along with morphology established the diagnosis. TRPS1 positivity in Müllerian tumors was also noted in previous studies. Rammal et al. found that 71% of endometrial cancers were TRPS1 positive [14]. This immunoreactivity limits the utility of TRPS1, particularly when Müllerian carcinoma is a diagnostic differential. In these settings, PAX8 and WT1 should be included in the diagnostic panel. We did not include cytology samples in our study. A study evaluated the utility of TRPS1 in the differential diagnosis of malignant pleural effusion found a high immunoreactivity of TRPS1 in metastatic carcinomas of tubo-ovarain origin/high grade serous carcinoma (75%) [15].

Although, in our study we did not have any skin/adnexal cancers, however previous studies demonstrated high TRPS1 expression across all skin and adnexal tumors. Cutaneous squamous and basal cell carcinomas had over 90% expression of TRPS1. Similarly, mucinous and sweat gland tumors (both benign and malignant) had high TRPS1 expression [13]. In addition, a variety of salivary gland tumors can rarely occur in breast and pose a significant diagnostic challenge. A recent study showed that approximately 92% of salivary gland carcinoma expressed TRPS1 [16]. Therefore, it should be kept in mind that in this context, where the differentials include a primary TNBC vs. a salivary gland malignancy, TRPS1 may not be helpful. Needless to say, GATA3 can also be positive in salivary gland tumors. Authors of the above-mentioned research emphasized the discriminatory role of SOX10 in this rare clinical context [16].

GATA3 positivity in urothelial carcinoma limits its usage when the differential diagnosis is bladder cancer. We also found TRPS1 positivity in a patient with metastatic urothelial carcinoma. Initial studies showed that TRPS1 was not expressed in bladder cancers and therefore, TRPS1 can be considered superior to GATA3 when the differentials include bladder and breast cancers [5]. However, more recent literature contradicts this opinion, as wider immunoreactivity of TRPS1 was unveiled. A recent study demonstrated that 24.6% prostatic adenocarcinoma and 20.5% urothelial carcinomas expressed TRPS1 [17]. These findings are supported by our study.

In addition to Mullerian, and bladder-origin tumors, TRPS1 positivity was also noted in 2 cases of lung origin tumors in our cohort. Data pertaining to immunoexpression of TRPS1 in lung cancers is scarce and variable. While the initial studies showed a low expression of TRPS1 in lung cancers, more recent studies demonstrated that a significant proportion of lung cancers showed TRPS1 expression. A study conducted on effusion specimens showed that 21.6% of metastatic lung cancers had TRPS1 immunoreactivity [18]. Expression of TRPS1 was also demonstrated in lung cancers at a molecular level [19]. These findings implicate that TRPS1 can not be used as a sole immunomarker to establish breast primary, however owing to its high expression across all breast cancer types it can be included in a panel to exclude or establish primary breast origin. A similar approach was suggested by Du et al., they suggested a combination of breast-specific markers including GATA3, TRPS1, and matrix Gla protein (MGP) as a diagnostic panel to establish breast primary [20].

### Limitations

The major limitation of this study is its retrospective design. Therefore, in some cases, not all immuno-markers were done. Moreover, the number of patients with non-breast primary was small, limiting the power of the statistical analysis. However, in all cases, the origin of the primary tumor was established by morphological correlation with IHC workup with molecular analysis in some cases (where necessary), which is a major study strength. In addition, we did not study TRPS1 expression at molecular/mRNA level or evaluated the prognostic significance of TRPS1 in breast cancers.

Another limitation of our study was that when TRPS1 positivity was noted in a metastatic sample, concurrent TRPS1 testing was not performed on the primary breast tumor sample. The diagnosis of metastatic carcinoma was made in the clinical context, with correlation of morphology, IHC profile and molecular studies (in a subset of cases).

We found a higher specificity of SOX10 in our cohort, compared to TRPS1. However, we did not include cases of primary or metastatic melanoma, which may overestimate this finding. As melanoma often mimics a poorly differentiated carcinoma, and can sometimes express cytokeratins, and therefore can be mistaken for a primary/metastatic carcinoma. Data regarding TRPS1 expression in melanoma is lacking. In our limited validation cohort (not included in the study cohort), we noted one case of melanoma with diffuse TRPS1 expression, however, a conclusive comment cannot be made without a large-scale study. In some of these difficult circumstances, with only SOX10 expression along with weak patchy cytokeratin staining, the only definitive solution remains molecular studies to identify UV/melanoma signature in the tissue sample. However, molecular studies are time-consuming, and therefore future studies to identify TRPS1 staining in melanoma can be very helpful. Moreover, GATA3 may be more helpful in this context, however GATA3 expression can be lost in poorly differentiated breast carcinoma, making situation more complex.

## Conclusions

Our study demonstrated a higher sensitivity of TRPS1 in establishing carcinoma of breast origin compared with GATA3 and SOX10, consistent with previous reported studies. TNBCs and metaplastic breast carcinoma, showed higher positivity of TRPS1, compared with GATA3 and SOX10. Conversely, the specificity of TRPS1 was lower than that of GATA3 and SOX10, limiting its utility.

Apart from breast, a broader immunoreactivity of TRPS1 was observed in non-breast tumors, including lung, Mullerian, and bladder cancers in our cohort. These findings imply that despite its higher sensitivity, TRPS1 cannot be used as a sole marker to establish primary breast origin. Moreover, as previous studies showed TRPS1 positivity in skin tumors, cartilage, and bone; therefore, its higher rate of positivity in metaplastic cancers may be due to squamous/mesenchymal differentiation rather than a marker of breast origin. While these findings also imply lack of utility of TRPS1 in differentiating metaplastic carcinoma vs. phyllodes tumor, more studies are required to prove this assumption.

In conclusion, we suggest that TRPS1 should only be used as part of a diagnostic panel, with either GATA3 or SOX10, depending upon site of biopsy and differentials.

## Data Availability

No datasets were generated or analysed during the current study.

## Abbreviations

AR: Androgen receptor
ER: Estrogen receptor
GATA3: GATA binding protein 3
GCDFP-15: Gross cystic disease fluid protein 15
HER2neu: Human epidermal growth factor receptor 2
IHC: Immunohistochemical
PR: Progesterone receptor
SOX10: SRY-related HMG box 10
TNBC: Triple negative breast cancer
TRPS1: Trichorhinophalangeal Syndrome Type 1
TTF1: Thyroid transcription factor 1
